# Optimum excitation wavelength and photon energy threshold for spintronic terahertz emission from Fe/Pt bilayer

**DOI:** 10.1016/j.isci.2022.104615

**Published:** 2022-06-16

**Authors:** Valynn Katrine Mag-usara, Mary Clare Escaño, Christopher E. Petoukhoff, Garik Torosyan, Laura Scheuer, Julien Madéo, Jessica Afalla, Miezel L. Talara, Joselito E. Muldera, Hideaki Kitahara, David R. Bacon, Makoto Nakajima, Keshav Dani, Evangelos Th. Papaioannou, René Beigang, Masahiko Tani

**Affiliations:** 1 Research Center for Development of Far-infrared Region, University of Fukui, Fukui 910-8507, Japan; 2 Institute of Laser Engineering, Osaka University, Suita, Osaka 565-0871, Japan; 3 Femtosecond Spectroscopy Unit, Okinawa Institute of Science and Technology Graduate University, Okinawa 904-0495, Japan; 4 KAUST Solar Center, Physical Science and Engineering Division, King Abdullah University of Science and Technology, Thuwal 23955-6900, Kingdom of Saudi Arabia; 5 Photonic Center Kaiserslautern, Kaiserslautern 67663, Germany; 6 Department of Physics, Technical University of Kaiserslautern, Kaiserslautern 67663, Germany; 7 Faculty of Pure and Applied Sciences, University of Tsukuba, Tsukuba 305-8573, Japan; 8 Institute of Physics, Martin Luther University of Halle-Wittenberg, Halle 06108, Germany

**Keywords:** physics, photonics, radiation physics

## Abstract

Terahertz emission from ferromagnetic/non-magnetic spintronic heterostructures had been demonstrated as pump wavelength-independent. We report, however, the pump wavelength dependence of terahertz emission from an optimized Fe/Pt spintronic bilayer on MgO substrate. Maximum terahertz generation per total pump power was observed in the 1200- to 1800-nm pump wavelength range, and a marked decrease in the terahertz emission efficiency beyond 2500 nm (pump photon energies <0.5 eV) suggests a ∼0.35-eV threshold pump photon energy for effective spintronic terahertz emission. The inferred threshold is supported by previous theoretical results on the onset energy of significant spin-filtering at the Fe-Pt interface, and confirmed by Fe/Pt electronic structure calculations in this present work. The results of terahertz time-domain emission spectroscopy show the sensitivity of spintronic terahertz emission to both the optical absorptance of the heterostructure and the energy-dependent spin transport, as dictated by the properties of the metallic thin films.

## Highlights

• Excitation wavelength dependence of THz emission from spintronic Fe/Pt bilayer
• Maximum THz emission efficiency of Fe/Pt in the 1200 to 1800-nm pump wavelength range
• Inferred pump photon energy threshold linked to spin-filtering at the Fe-Pt interface
• Effect of pump absorptance and energy-dependent spin transport on Fe/Pt THz emission

## Graphical abstract

**Figure:**
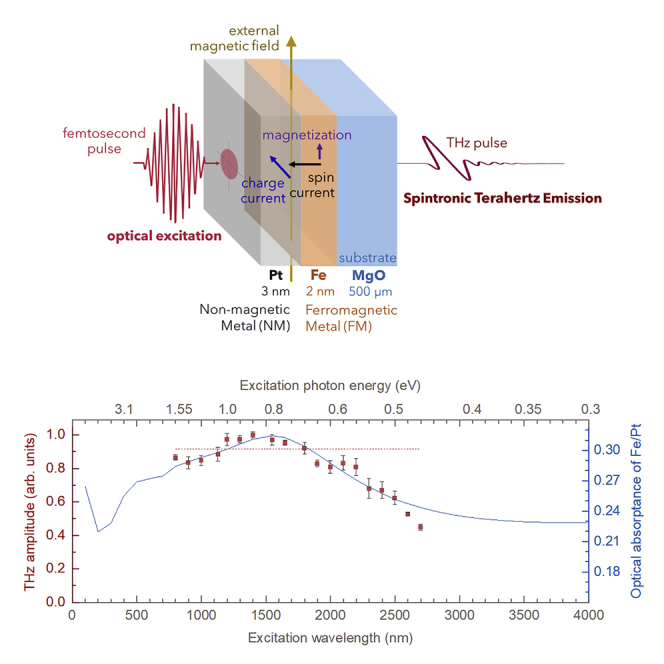

## Introduction

In recent years, an innovation in terahertz (THz) science and technology materialized when a fairly efficient spintronics approach in generating broadband terahertz (THz) radiation was demonstrated by conveniently illuminating magnetic heterostructures with ultrafast laser pulses (Kampfrath et al., 2013). Such magnetic heterostructures basically consist of ultrathin ferromagnetic (FM) and non-ferromagnetic (NM) metallic layers. The emergence of these FM/NM-based metallic spintronic heterostructures as THz sources has allowed both prominent fields of spintronics and THz science to mutually benefit from each other with their convergence in THz spintronics (Walowski and Münzenberg, 2016; Papaioannou and Beigang, 2021). From the perspective of THz research, spintronic THz emitters constitute a desirable new class of THz sources, which are particularly attractive to develop due to their promising advantages in terms of versatility, durability, ease of operation, high pump-power scalability, and broadband THz emission with tunable polarity (Kampfrath et al., 2013; Walowski and Münzenberg, 2016; Papaioannou and Beigang, 2021; Seifert et al., 2016; Huisman and Rasing, 2017; Yang et al., 2016; Wu et al., 2017; Torosyan et al., 2018; Papaioannou et al., 2018). They do not require electrical contacts nor external voltage bias to generate THz radiation and only need relatively weak in-plane magnetic bias to establish the magnetic order in their FM layers (Torosyan et al., 2018). The spintronic THz emission mechanism (Kampfrath et al., 2013) exploits the spin property of electrons and is remarkably distinct (Walowski and Münzenberg, 2016; Papaioannou and Beigang, 2021) from those exhibited by other laser-driven THz sources (Lee, 2009; Lewis, 2014), such as nonlinear optical crystals like ZnTe and GaP (Han and Zhang, 1998), semiconductor surface emitters like InAs and InSb (Gu et al., 2002), air plasma (Kress et al., 2004), and photoconductive antennas (PCA) (Tani et al., 1997).

The generation of THz radiation in metallic spintronic heterostructures with FM/NM layers occurs as a result of a two-part process in which an ultrafast laser pulse-induced spin current from an in-plane-magnetized FM layer is effectively converted by inverse spin-Hall effect (ISHE) (Saitoh et al., 2006) into a transverse charge current in the NM layer, due to strong spin-orbit interaction (Kampfrath et al., 2013; Walowski and Münzenberg, 2016; Papaioannou and Beigang, 2021; Seifert et al., 2016; Huisman and Rasing, 2017; Yang et al., 2016; Wu et al., 2017; Torosyan et al., 2018; Papaioannou et al., 2018). In the first part, spin current is generated in the FM layer upon optical excitation, when the energy of the femtosecond (fs) laser pulse causes an out-of-equilibrium electron distribution in the ferromagnetic material. Due to asymmetries in the lifetimes and velocities of majority-spin and minority-spin electrons (Zhukov et al., 2006; Battiato et al., 2012), this non-equilibrium condition immediately sets up the sub-picosecond timescale transport of spin-polarized electrons from the FM layer. In the second part, ISHE transforms the spin current into charge current upon spin current injection to the NM layer through the FM-NM interface. The presence of strong spin-orbit coupling causes the spin-polarized electrons to deviate from their path and follow opposite directions according to their spin orientations. The resulting transverse transient charge current, which is perpendicular to both the spin current direction and spin polarization direction, consequently emits THz radiation into the optical far-field. The spin-to-charge current conversion mechanism obeys

(Equation 1) $$j_{c}={\text{θ}}_{\text{SH}}j_{s}\times \sigma$$

where ***j***_***c***_ is the charge current, θ_SH_ is the spin-Hall angle and a measure of the electron deflection, ***j***_***s***_ is the spin current, and ***σ*** is the spin polarization vector, which is parallel to the sample magnetization direction (Kampfrath et al., 2013; Saitoh et al., 2006). The THz emission, which is proportional to the time derivative of the charge current, exhibits polarization that depends only on the magnetization direction, sign of the spin-Hall angle, and the properties of the FM and NM layers (Kampfrath et al., 2013; Walowski and Münzenberg, 2016; Papaioannou and Beigang, 2021; Seifert et al., 2016; Huisman and Rasing, 2017; Yang et al., 2016; Wu et al., 2017; Torosyan et al., 2018).

The successful demonstration of THz generation due to spin-to-charge current conversion in Fe/Au and Fe/Ru magnetic heterostructures (Kampfrath et al., 2013) was replicated and explored further by other research groups using various combinations of FM and NM materials in bilayer, trilayer, and multilayer heterostructures. The published works to-date include investigations on optimum layer thicknesses (Seifert et al., 2016; Yang et al., 2016; Wu et al., 2017; Torosyan et al., 2018; Zhang et al., 2018; Qiu et al., 2018a; Kumar et al., 2021), material choices for substrates (Wu et al., 2017; Torosyan et al., 2018; Nenno et al., 2019), incorporation of liquid crystal (Qiu et al., 2018b), dielectric cavity (Herapath et al., 2019), striped patterns (Yang et al., 2016; Jin et al., 2019), and antenna structures (Nandi et al., 2019; Talara et al., 2021); different growth parameters (Torosyan et al., 2018; Nenno et al., 2019), defect engineering (Nenno et al., 2019), and optical damage limit (Kumar et al., 2021). Several of these investigations have Pt as the NM material (Seifert et al., 2016; Yang et al., 2016; Wu et al., 2017; Torosyan et al., 2018; Qiu et al., 2018a; Qiu et al., 2018b; Herapath et al., 2019; Jin et al., 2019; Nandi et al., 2019; Nenno et al., 2019; Matthiesen et al., 2020; Talara et al., 2021; Kumar et al., 2021). The choice of NM material is critical because it primarily influences the amplitude and polarity of the THz emission (Seifert et al., 2016). Platinum, with its high spin-Hall conductivity (Hoffman, 2013), is a good non-ferromagnetic metal thin layer for a metallic spintronic heterostructure-based THz emitter.

**Figure 1:**
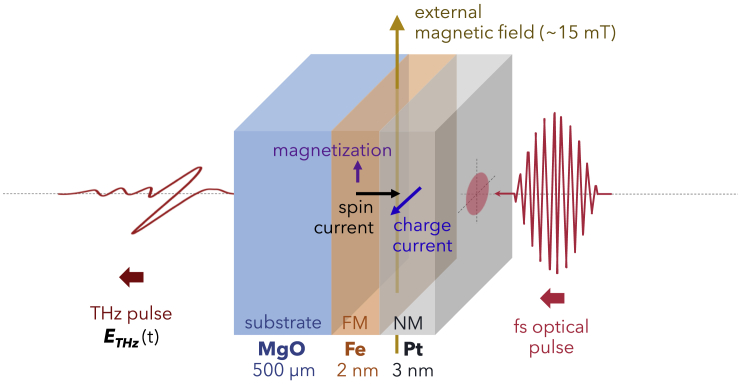
The configuration for spintronic THz generation using the optimized Fe/Pt bilayer Fe and Pt are the ferromagnetic (FM) and non-ferromagnetic (NM) metallic thin films, respectively. Note that the dimensions are not drawn to scale.

Spintronic THz emission of an optimized Fe/Pt bilayer structure has already been the subject of a series of investigations (Torosyan et al., 2018; Papaioannou et al., 2018; Nenno et al., 2019; Mag-usara et al., 2020). The ideal thickness combination of epitaxial Fe and Pt metallic thin films for THz emission was experimentally determined in qualitative agreement with simulations of induced spin current in the Fe layer, wherein the theoretical model took into account the generation of spin polarization, spin diffusion and accumulation in the layers, as well as the electrical and optical properties of the bilayer (Torosyan et al., 2018). The optimized spintronic bilayer consists of 2-nm Fe and 3-nm Pt which were epitaxial grown on a 1 × 1 cm^2^ piece of 500-μm thick MgO substrate. This Fe/Pt-based THz source exhibited the favorable properties of metallic spintronic heterostructures intended for laser-driven broadband THz generation and it was reported with high optical damage threshold (above 5 mJ/cm^2^). In the previous studies (Torosyan et al., 2018; Papaioannou et al., 2018; Mag-usara et al., 2020), the properties of this Fe/Pt spintronic bilayer were investigated by THz time-domain emission spectroscopy, using either a femtosecond fiber laser or a mode-locked Ti:sapphire fs oscillator laser to provide the pump and probe pulses. The optimized bilayer proved to be a versatile THz radiation source, as it exhibited fairly the same THz emission efficiency per average pump power at 400 (3.0 eV), ∼800 (1.55 eV), and 1550-nm (0.8 eV) excitation wavelengths, even with relatively low average excitation power and even though the pump photon energies are significantly different (Papaioannou et al., 2018; Mag-usara et al., 2020). Another group, which studied THz generation in W/CoFeB/Pt trilayer, demonstrated that the spintronic THz emission is wavelength-independent in the 900- to 1500-nm optical excitation range (Herapath et al., 2019). Collectively, the observations using the Fe/Pt bilayer and the W/CoFeB/Pt trilayer imply that, in the investigated pump wavelength region, the important factor in spintronic THz generation is not the photon energy of the optical pump pulses but their total energy input (pump photon energy multiplied with the number of photons) which drives the spin-polarized electron transport in the metallic heterostructure (Papaioannou et al., 2018; Herapath et al., 2019; Mag-usara et al., 2020). While these independent investigations on a bilayer and a trilayer are consistent with each other, the wavelength-independent picture of spintronic THz emission is far from complete. The influence of even longer pump wavelengths (above 1550 nm) on spintronic THz emitter operation has not been explored. It was postulated that the spintronic THz generation efficiency is expected to be constant for sufficiently large excitation photon energies and will be different with excitation by very low energy photons (Papaioannou et al., 2018). However, that statement was presented without experimental validation and the scenario of THz generation at significantly lower photon energies remains unclear. Hence, in this report, we deal with the optimized 2-nm Fe/3-nm Pt bilayer and employ THz time-domain emission spectroscopy to further probe how THz generation in this spintronic heterostructure is influenced by optical excitation in the 800- to 2700-nm wavelength range. For this study, the spintronic THz emitter was irradiated with pump pulses from a wavelength tunable femtosecond laser system consisting of a Ti:sapphire regenerative amplifier operated in tandem with an optical parametric amplifier. The magnetization direction in the ferromagnetic layer was controlled by a ∼15-mT external magnetic field oriented along the plane of the Fe film and perpendicular to the direction of the pump pulses, as illustrated in Figure 1. The detailed description of the experiment and measurement system is provided in the STAR Methods Section and illustrated in Figure S1 of the Supplemental Information. The THz radiation from the Fe/Pt was found to exhibit pump wavelength dependence, which was more pronounced at wavelengths longer than 2200 nm (pump photon energies below ∼0.56 eV) and indicated a pump photon energy threshold at ∼0.35 eV. In addition, the optimal THz emission efficiency, which is the THz emission per incident pump power, was observed to be within the 1200- to 1800-nm pump wavelength range.

## Results and discussion

**Figure 2:**
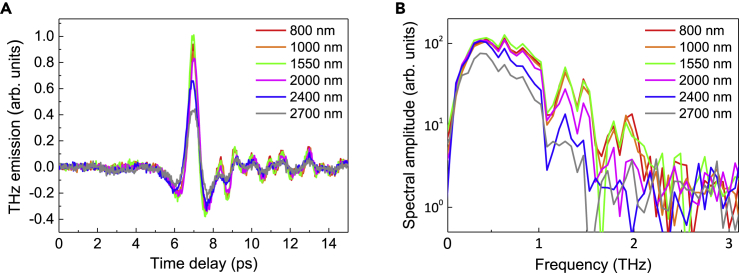
Representative waveforms and spectra of the THz emission from the Fe/Pt spintronic bilayer The Fe/Pt on MgO spintronic THz emitter was optically pumped at a fixed irradiation spot using 5-mW average pump power and ∼4-mm diameter beam spot size. With optical excitation from 800 to 2700 nm, the shape of the (A) THz time-domain waveforms and (B) corresponding spectra of the THz emission remained relatively unchanged. The observable change is in terms of the THz emission amplitude particularly when the pump wavelengths are longer than 2200 nm.

The THz generation in the spintronic bilayer was evaluated at nineteen (19) different pump wavelengths within the 800- to 2700-nm range without changing the pump beam pointing on the Fe/Pt bilayer, the overall alignment, and other key parameters of the experiment. The average power and spot size of the collimated pump beam that was incident on the Fe/Pt emitter were also maintained at 5 mW and 4-mm diameter, respectively, such that the pump fluence remained constant on the same spot at ∼40 μJ/cm^2^ as we tuned the laser source to generate pump pulses at target wavelengths. In Figure 2A, representative waveforms of the spintronic THz emission generated by different pump wavelengths show that the shapes of the THz signals and their corresponding spectra (Figure 2B) remained relatively the same even as the pump wavelengths significantly changed. The observable change is in terms of the THz amplitude, which decreased considerably when the wavelengths for the optical excitation of the Fe/Pt spintronic emitter were already above 2200 nm. The time-domain waveforms in Figure 2A feature oscillations appearing after the main single cycle pulse, and their corresponding frequency spectra (Figure 2B) feature prominent dips at specific frequencies. Such features are attributed to water vapor absorption, as the THz emission measurements were carried out under ambient room condition with a relative humidity of 55 ± 0.5%. The oscillations in time domain are ascribed to water absorption and re-emission of THz waves by free induction decays. In Figure 2B, the water absorption bands (van Exter et al., 1989) may not be so sharp due to the limited time window of the time-domain spectroscopy scan but they are particularly strong at ∼1.1 THz and ∼1.6–1.7 THz, and relatively weak but still observable at 0.6, 0.75, and 1.4 THz frequencies. It is apparent that the spectral background and the spectral peak position of the detected THz waves fluctuate due to such spectral noises. Despite the background, the main single cycle THz waveform is consistent under different pump wavelengths and the peak-to-peak signal is robust to the noises. Hence, the peak-to-peak measurement of the THz waveform is used as the indicator for the THz emission strength.

**Figure 3:**
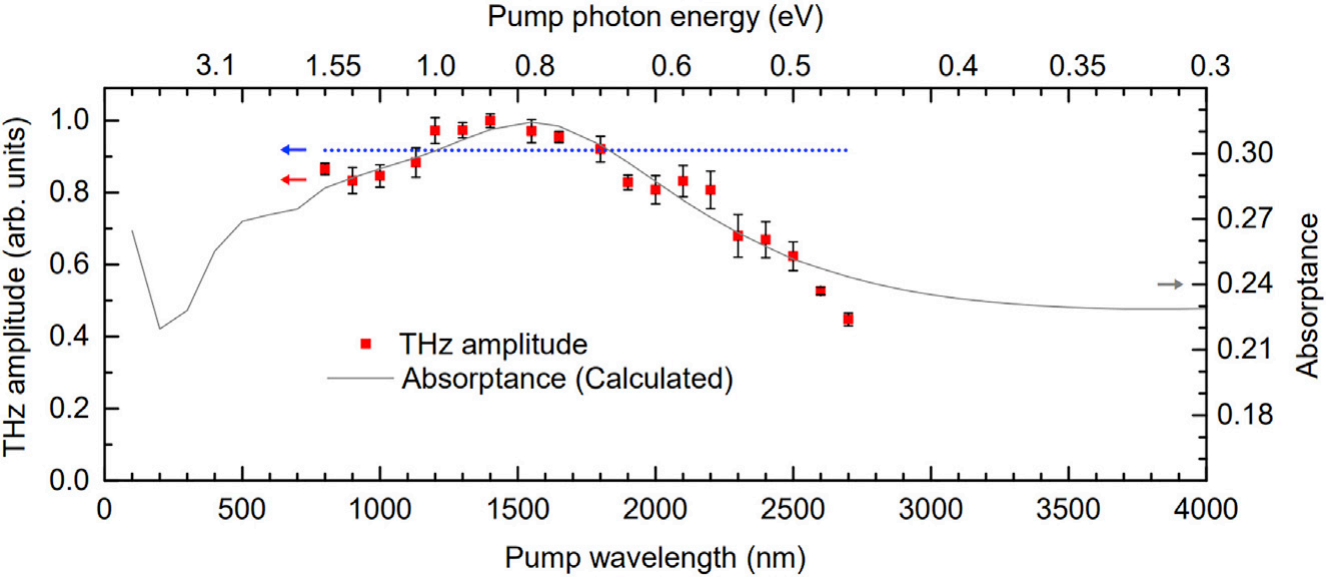
Plots of the peak-to-peak amplitudes of the spintronic THz emission time-domain waveforms and the calculated absorptance of the Fe/Pt bilayer The THz peak-to-peak amplitudes in the time-domain when the Fe/Pt is optically excited by near-infrared pump pulses with wavelengths from 800 to 2700 nm (or from 1.55 eV down to 0.46 eV in corresponding photon energy values) are represented by solid red squares. The dotted blue line represents the approximate THz amplitude level if the spintronic THz emission remained completely independent of the pump wavelength. For each pump wavelength or its pump photon energy equivalent, an error bar represents the largest absolute deviation of peak-to-peak THz amplitudes from the mean of six data points. The gray solid line represents the calculated optical absorptance of the Fe/Pt bilayer.

The pump wavelength and corresponding pump photon energy dependence of the spintronic THz emission are presented in Figure 3, where the peak-to-peak amplitudes of the THz time-domain waveforms are plotted as red solid squares. The dotted blue line represents the approximate THz amplitude level if the spintronic THz emission remained completely independent of the pump wavelength, as observed in the previous studies on optical excitation wavelengths and THz emission from metallic heterostructures (Papaioannou et al., 2018; Herapath et al., 2019; Mag-usara et al., 2020). Here, the dotted blue line is plotted based on the average of the peak-to-peak amplitudes of the Fe/Pt bilayer’s THz emission from 800 to 1550 nm, taking into consideration that those studies, which investigated spintronic THz generation by pump wavelengths only up to 1550 nm, yielded corroborating results on the wavelength independence of spintronic THz emitters. Figure 3 shows that, for the aforementioned wavelength range, the variation in THz emission efficiency of the 5-nm thick Fe/Pt bilayer is within 20% (i.e. ∼17%), which is not so different from the ∼15% variation observed in THz generation using a 5.8-nm thick W/CoFeB/Pt spintronic trilayer in the 900 to 1500-nm range (Herapath et al., 2019). However, this present work also shows that the previously reported wavelength-independent characteristic of the spintronic THz emission does not hold especially at longer pump wavelengths or lower pump photon energies. It is clear that the THz radiation per incident pump power of the Fe/Pt metallic heterostructure exhibits the influence of the pump wavelength.

Over a wide range of optical excitation wavelengths, from 800 to 2200 nm, the spintronic THz emission seems to have no strong pump wavelength dependence. Even so, it can also be easily surmised from the data in Figure 3 that the Fe/Pt bilayer is most optimized for THz generation using pump pulses having wavelengths in the 1200- to 1800-nm range (∼0.7 eV–1.0 eV), where up to 15% more THz emission per incident pump power can be obtained than by using the 800-nm pump that was commonly utilized in the previous studies. This confirms the versatility of the spintronic emitter as a THz source and that the Fe/Pt on MgO is ideal for integration in low-cost, compact THz systems driven by femtosecond fiber lasers operating at around 1550 nm (Papaioannou et al., 2018). The better THz emission efficiency exhibited by the bilayer with 1550-nm optical excitation, even if it is just ∼10% higher compared with that by the 800-nm pump, is noteworthy and has commercial merit especially when considering the performance of conventional pulsed laser-driven THz emitters at the same pump wavelengths. Commonly used THz sources which have reliable emission efficiency with 800-nm optical excitation, such as the LT-GaAs PCAs and nonlinear optical crystals like ZnTe, are expensive but do not perform as well when optically excited by 1550-nm pump pulses. The THz emission efficiency of LT-GaAs PCA with 1550-nm excitation is just ∼10% of that by 800-nm pump pulses (Tani et al., 2000; Mag-usara et al., 2020). In addition, the efficiency of THz generation by optical rectification of femtosecond laser pulses in nonlinear crystals is limited by the phase mismatch or velocity mismatch between the optical pump and the THz waves, especially for 1550-nm pump wavelength where nonlinear crystal with good phase-matching condition has not been found so far. In such cases, these conventional THz sources which work well with 800-nm pump would have to be replaced entirely with emitters that are specifically for 1550-nm operation. However, InGaAs-based PCAs, which have photoconductive energy bandgap that is well matched to the photon energy of 1550-nm excitation, are much more expensive than LT-GaAs PCAs but have low resistance and cannot achieve a similar level of efficiency as that of LT-GaAs PCAs pumped at 800 nm. The advantage of spintronic THz emitters like Fe/Pt is ease of use, since they do not require special and costly adjustments. Particularly, for application under 1550-nm optical excitation, the THz emission efficiency of Fe/Pt highlights further advantage due to its high optical damage threshold which allows for the THz emission to be increased significantly by simply using high pump fluence.

In Figure 3, the plot of the calculated absorptance of the Fe/Pt bilayer is also shown. By assessing the optical absorptance, which is the ratio of absorbed optical power to the incident optical power, the pump light absorption in the spintronic THz emitter can be taken into account. Consequently, the influence of the pump wavelength on the spintronic THz emission can be properly elucidated in terms of the absorbed laser power in the metallic heterostructure. The absorptance values from 100 to 4000 nm were obtained based on the appropriate relevant formulae (Tomlin, 1972; Barybin and Shapovalov, 2010) for the double thin film structure of the Fe/Pt that is bounded by ambient air on the Pt side and by 500-μm MgO substrate on the Fe side. The calculations, which take into consideration that the pump beam enters the metallic heterostructure at normal incidence by penetrating the 3-nm Pt film first before reaching the 2-nm Fe, are described further in the STAR Methods Section.

**Figure 4:**
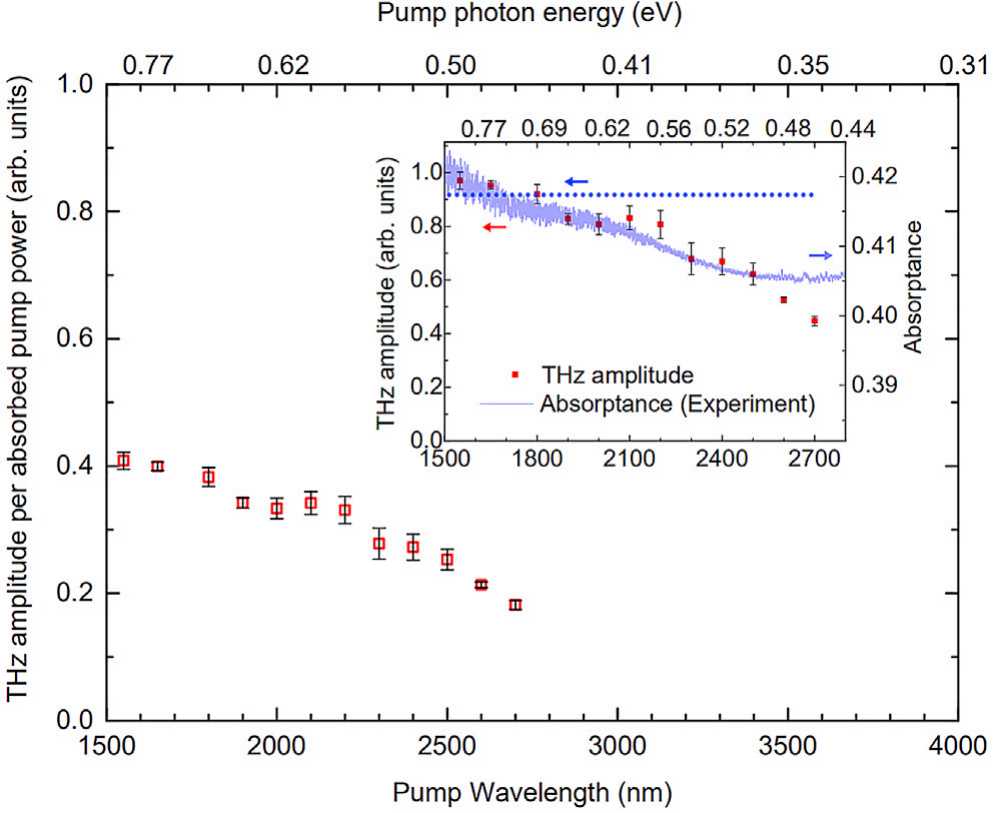
THz emission amplitude per absorbed pump power of the 2-nm Fe/3-nm Pt bilayer The open squares represent the peak-to-peak amplitudes of the THz emission when the corresponding absorptance (deduced from Fourier-transform infrared or FTIR spectroscopy) of the Fe/Pt are taken into account with the pump wavelengths or photon energies. Each error bar represents the largest absolute deviation of peak-to-peak THz amplitude per absorbed pump power from the mean of six data points for each pump wavelength or pump photon energy. Inset: Comparison plot of the measured THz emission (solid red squares with error bars, as in Figure 3) and the Fe/Pt absorptance curve (purple solid line) generated from experiments. The dotted blue line represents the approximate THz amplitude level if the spintronic THz emission of the bilayer remained invariant with the pump wavelength. The experimentally determined absorptance vs. pump wavelength (and corresponding photon energy) is limited from 1500 nm by the range of the deuterated L-alanine-doped triglycine sulfate (DLaTGS) detector used in the FTIR measurements of transmittance and reflectance.

It can be easily deduced from the plots in Figure 3 that the THz emission exhibits sensitivity to the optical absorption efficiency of the spintronic thin-film heterostructure. The observed slight enhancement of the spintronic THz emission in the 1200- to 1800-nm wavelength range can be ascribed to the optimal optical absorption in the FM/NM bilayer since the absorptance of the 2-nm Fe/3-nm Pt has its maximum within this wavelength range. The trend of the THz emission amplitudes of the Fe/Pt bilayer with respect to the pump wavelength and the pump photon energy is generally consistent with that of the calculated absorptance except at the pump wavelengths longer than 2500 nm or at photon energies below 0.5 eV. This is also similarly observed in the inset of Figure 4 with respect to the absorptance deduced from measurements of the Fe/Pt transmittance and reflectance by cw Fourier transform infrared (FTIR) spectroscopy. The dataset of absorptance based on actual experiments is limited because we do not have the appropriate detector that would allow decent signal-to-noise ratio for FTIR measurements between 1100 and 1500 nm. Nevertheless, the trend of the absorptance shown in the Figure 4 inset is qualitatively consistent with the calculated absorptance for the same range. The experimentally obtained absorptance is approximately 10% higher than the calculated dataset and varies by no more than 2% in the 1500-nm to 2800-nm range but, upon closer scrutiny, it also has the same decreasing trend as that of the THz amplitudes, except for above 2500 nm. We note that metals and semimetals generally exhibit transient reflectivity changes during the first ps of pulsed laser irradiation and it might be argued that these would significantly affect the absorptance. However, such changes are actually very small. As an example, for a pump fluence of 2.5 mJ/cm^2^ on Pt film, the transient reflectivity induced by either 800-nm or THz pump pulses only changed by a maximum of <0.15% (Unikandanunni et al., 2022). In this work, the pump fluence was only ∼40 μJ/cm^2^ for all wavelengths, thus the corresponding transient reflectivity changes for each pump wavelength should be very small as well and would have negligible influence on the optical absorptance.

The influence of pump light absorption in the Fe/Pt bilayer to its THz emission is accounted for in Figure 4, which shows the THz emission amplitudes normalized by the absorptance based on FTIR measurements. Consistent with the observation in Figures 3 and 4 inset, where the THz amplitudes are shown without absorption correction, the THz emission per absorbed pump power shown in Figure 4 exhibits relative invariance to the pump wavelengths up to 2200 nm (∼0.56 eV). It is clear in both Figures 3 and 4 that the THz emission of the Fe/Pt heterostructure per total optical input power is limited by the absorptance, which is below 50% even at the maximum. The unfavorable impact of the relatively low pump light absorption to the THz emission efficiency is even more pronounced with optical excitations using low photon energies, considering that the absorptance approaches a minimum below 0.5 eV. It is an interesting observation, however, that despite the remarkable sensitivity of the spintronic THz emission to the absorptance of the Fe/Pt, it decreases significantly and markedly deviates from the absorptance trend when the pump photon energies fall below 0.5 eV. Both figures show that the THz emission amplitude decreases much faster than the rate at which the absorptance curves approach their minima. The deviation implies that the substantial reduction in the spintronic THz emission in the sub-0.5 eV photon energy regime cannot be attributed to the decrease in absorptance and there is a mechanism that adversely influences the spintronic THz emission of the Fe/Pt bilayer in the low photon energy region.

The results, as presented in Figures 3 and 4, provide experimental evidence to the conjecture that the spintronic THz generation efficiency is constant for sufficiently large excitation photon energies but will be different for low photon energies (Papaioannou et al., 2018). From both figures, it can also be inferred even by visual extrapolation that, if the trend of considerable decrease in THz emission amplitude continues, the THz emission of the 2-nm Fe/3-nm Pt bilayer would have an onset at photon energies within the 0.3 to 0.45 eV range. The trend of the THz amplitude per absorbed pump power in Figure 4, in particular, suggests that the pump photon energy threshold of substantial THz emission using the optimized Fe/Pt heterostructure is ∼0.35 eV. Since the THz emission of the Fe/Pt spintronic heterostructure arises from the spin current generation in the Fe layer and then by spin current injection from the Fe to the Pt layer, the inference on an onset photon energy for effective spintronic THz emission is very closely linked to these two requisite processes of the THz generation mechanism in the bilayer.

**Figure 5:**
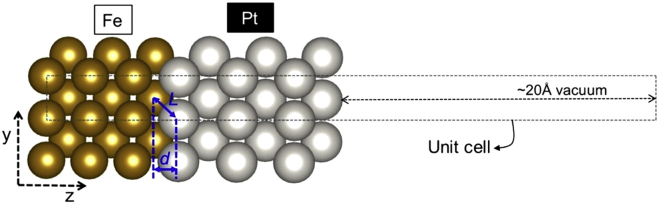
Model structure of Fe/Pt bilayer laid along z axis The bilayer is composed of six layers Fe(001) and six layers of pseudomorphic Pt(001) slab. Due to symmetry along xy plane, (1x1) surface unit cell is sufficient and is shown in dashed lines. A ∼20 Å of vacuum is introduced in the unit cell to separate the slab from that of the repeated unit cell along z direction. Fe and Pt sides are labeled accordingly and the interface bond and the interlayer distances along z are depicted as *L* and *d*, respectively.

A recent theoretical study on spin- and energy-dependent reflectivity at the Fe/Pt bilayer interface reported a marked decrease of spin-up reflectivity $\left(R_{Fe\to Pt}^{↑}\right)$ at 0.25 eV above the Fermi level (E_F_), while that of the spin-down $\left(R_{Fe\to Pt}^{↓}\right)$ remains the same, indicating significant spin-filtering (i.e. difference between $R_{Fe\to Pt}^{↑}\text{ and }R_{Fe\to Pt}^{↓}$ and, therefore, a significant spin injection from Fe to Pt (Lu et al., 2020). Note that this onset energy of 0.25 eV for effective spin injection is close to the pump photon energy threshold inferred from the experimental results. To confirm this conjecture and to directly correlate it to the material’s electronic properties, we also conducted electronic structure calculations of Fe/Pt. Since the markedly low spin-up reflectivity from 0.25 eV was attributed to a low spin-up Sharvin conductance of Fe $\left(G_{SH,Fe}^{↑}\;\right)$ (Lu et al., 2020) and, hence, low propagating 3*d*-states of Fe, we calculated the spin-up and spin-down 3*d*-states of the Fe side of the bilayer (i.e. local density of states or LDOS) using spin-polarized density functional theory (SDFT) (Hohenberg and Kohn, 1964; Kohn and Sham, 1965) and note the changes in the spin-up 3*d*-states corresponding to the decrease in the reflectivity. As illustrated in Figure 5, the atomic model for the Fe/Pt bilayer is formed by a (1x1) surface unit cell consisting of six layers of Fe(001), six layers of Pt(001), and a vacuum of ∼20 Å. This model slab is sufficient to include the interface and the bulk of Fe. The details of the SDFT calculation method and the tests conducted for its suitability in the treatment of atomic, electronic, and magnetic structures of Fe and Pt are provided in the STAR Methods Section.

**Figure 6:**
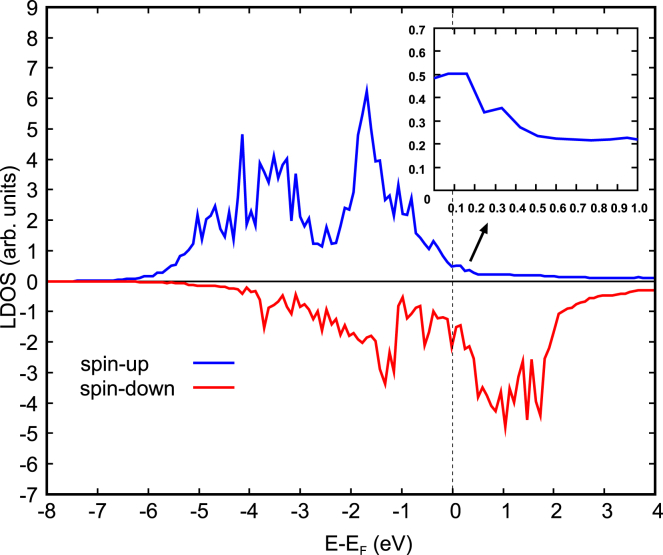
Local density of states (LDOS) of Fe, where the *d*-states are shown LDOS is plotted for the Fe atoms at the interface down to the bulk (middle portion of the Fe side). Spin-up and spin-down states are indicated in blue and red lines, respectively. The inset is a close-up view of the section of spin-up LDOS around 0 to 1.0 eV.

The calculated LDOS is shown in Figure 6, where we can easily see the very high values of the spin-down LDOS in comparison to that of the spin-up, which confirms the constantly very high spin-down reflectivity that was reported (Lu et al., 2020). Moreover, the LDOS clearly shows that the spin-up *d*-states of Fe significantly decrease from the E_F_ to energies above E_F_. Specifically, there is a marked decrease in electron states from 0 to 0.25 eV as shown in the Figure 6 inset (the enlarged section of the LDOS around 0 to 1.0 eV), which matches the energy of decrease in spin-up reflectivity (Lu et al., 2020) and would explain the inferred onset of substantial spintronic THz emission (∼0.35 eV) in this present work. It can be clearly observed that the spin-up LDOS eventually flattens at very low values but, within 0.25–0.5 eV above the E_F_, it only starts to continuously gradually decrease from ∼0.35 eV. From this point in energy onward, the spin-filtering efficiency in Fe/Pt is improved because of the also higher spin-down LDOS states. Hence, the inferred onset of substantial spintronic THz emission (∼0.35 eV) is found to be consistent with the LDOS, which also validates the previous results (Lu et al., 2020) of first-principles calculations on the energy-dependent spin transport in Fe/Pt. Interestingly, the correlation of the LDOS of the ferromagnetic material and the reflectivity across the interface is also consistent with the investigation on Co/Pt (Dang et al., 2020). Briefly, for Co/Pt, the spin-up transmission is high and constant from 0 to 1 eV due to very little spin-up LDOS in this region while the spin-down transmission is low because of the relatively high spin-down LDOS. Such correlation on the electronic structure of FM/NM with the reflectivity (or transmission) at the interface can be expected because of the localized nature (low group velocity) of 3*d*-states. When these states decrease, they give way to the propagation of the more delocalized 4*s* electrons.

Through SDFT calculations, we have confirmed that the experimental results also demonstrate the fundamental influence of energy-dependent spin transport on spintronic THz generation. The optical pump wavelength dependence of the measured emission exhibits the sensitivity of the THz generation process not only to the absorptance of the spintronic heterostructure but also to both spin current generation in the FM layer and the subsequent FM→NM spin current injection. Such sensitivity is particularly more obvious in the low photon energy excitation region, where we inferred a threshold pump photon energy for effective THz emission from the Fe/Pt bilayer.

We clarify that the spin current ***j***_***s***_ in Equation 1 is the spin current from the FM layer, ***j***_***s***_^***FM***^ that reaches the NM layer, such that ***j***_***s***_ = ***j***_***s***_^***NM***^. The relationship between ***j***_***s***_^***FM***^ and ***j***_***s***_^***NM***^ is governed by the efficiency of spin current injection, which is the spin-polarized electron transport through the FM-NM interface. This can be summarized as $j_{s}^{NM}=\;\xi \left(E\right)j_{s}^{FM}\text{,}$ where ξ(E) is the spin current injection coefficient that is influenced by the spin- and energy-dependent interface reflectivity. Upon irradiating the spintronic heterostructure by ultrafast laser pulses, absorbed photons generate spin current in the FM by exciting spin-polarized electrons. The spin-polarized electrons superdiffuse at the FM-NM interface with spin- and energy-dependent reflectance. The interface dictates the efficiency of the spin current injection process. Thus, to optimize the THz generation process across a wide range of optical excitation wavelengths, spintronic THz emitter devices have to be designed such that the properties of the FM, NM, and the interface not only allow for ideal optical pump absorption but also facilitate the best possible energy-dependent spin transport that leads to spin-to-charge current conversion at target operation wavelengths.

We have, so far, discussed the optical pump absorptance and energy-dependent spin transport in the optimized Fe/Pt bilayer, which are indeed important to consider because the efficiency of THz generation using a spintronic heterostructure is dependent on three inter-related and sequential processes: optical excitation, spin current generation and spin transfer to the Pt layer, and the generation of transient charge current due to spin-to-charge current conversion in the Pt layer. The efficiency of optical excitation can be assessed through the pump absorptance of the heterostructure while the efficiency of spin current generation and transfer can be predicted from the LDOS and energy-dependent spin transport. If the optical excitation and spin transport efficiency are both low to begin with, such as in the low photon energies, then the next process that leads to THz emission would already be compromised. All three processes essentially work together and they are challenging to uncouple when the information is only THz emission.

There is merit in investigating the THz generation from other bilayer or multilayer spintronic heterostructures using different pump wavelengths in order to elucidate the optical excitation wavelength or pump photon energy at which their respective THz emission efficiencies start decreasing and to confirm if such spintronic heterostructures also have pump photon energy thresholds. Different FM and NM have their own electronic properties, which also influence the properties of their various combinations. For example, based on the calculated interface reflectivities of Fe/Al (Lu et al., 2020), the spin-filtering which influences the spin injection from Fe to Al starts to decrease with photon energy at ∼0.6 eV from the Fermi level, as indicated by the onset of narrowed difference between the spin-up and spin-down reflectivities. Ni/NM heterostructures are also interesting because, unlike the Fe-NM interfaces, the Ni-NM interfaces do not act as spin filters but the THz emission of Ni/NM heterostructures is still expected to be substantial (Lu et al., 2020). Other thickness combinations of Fe and Pt may be explored to directly corroborate the observations of pump wavelength dependence due to pump absorptance in this work. Pump wavelength dependence measurements of the spintronic THz emission can also be utilized to probe other properties, such as the optical damage limit (Kumar et al., 2021) of various spintronic heterostructures.

### Conclusions

In summary, we have shown that the THz emission from a metallic spintronic heterostructure, such as the optimized Fe/Pt bilayer on MgO substrate, is not totally independent of the optical excitation wavelength due to optical absorptance and spin-filtering. While the Fe/Pt bilayer is indeed a versatile THz source as its performance remains fairly the same over a wide range of pump wavelengths, it actually exhibits a slight enhancement of the spintronic THz emission efficiency in the 1200- to 1800-nm pump wavelength range and such efficiency continuously decreases when the excitation wavelength goes further beyond 2200 nm. The observed influence of the optical pump wavelength on the THz emission which, at wavelengths longer than 2500 nm, is inconsistent with the absorptance, led us to infer a ∼0.35-eV threshold pump photon energy for effective spintronic terahertz generation in the Fe/Pt bilayer. The threshold can be ascribed to the onset of significant spin-filtering in the Fe/Pt bilayer. Our experimental and theoretical results are consistent with information based on first-principles calculations on the energy-dependent spin transport. Therefore, in studying the wavelength dependence of THz radiation from a metallic spintronic heterostructure by THz emission spectroscopy, we were able to demonstrate the influence of both optical pump absorptance and energy-dependent spin transport on the spintronic THz emission process. While previous studies showed that spintronic THz emitters are pump wavelength-independent, this work with a 2-nm Fe/3-nm Pt bilayer demonstrated otherwise and explained why in terms of optical absorptance and spin-filtering.

### Limitations of the study

The spintronic THz emission experiments with different pump wavelengths were carefully designed to guarantee the proper comparison of results by keeping all the other parameters the same as much as possible. The experimental conditions for THz generation at different optical excitation wavelengths using the optimized Fe/Pt bilayer and THz detection using a 1-mm thick ZnTe electro-optic crystal were maintained for all measurements. The properties of the collimated output beam of the optical parametric amplifier (OPA) used in our experiments were very well taken into consideration to ensure that the beam incidence and beam quality on the spintronic THz emitter remained unchanged for all target wavelengths, with no distortion nor chromatic aberrations complicating the results. The fluence, spot shape, spot size, and position of the pump beam on the emitter surface were kept the same. The incident pump beam was intentionally kept collimated and never focused onto the Fe/Pt heterostructure. The fluence, which was well below the damage limit of the 2-nm Fe/3-nm Pt bilayer, was also reasonably chosen to ensure that the quality and the layer thicknesses of the heterostructure remained constant for all measurements. It was confirmed both by autocorrelation and spectral measurements that the output beam of the OPA consistently exhibited Gaussian properties, which also means that the transverse profile of the collimated excitation beam’s optical intensity remained Gaussian. The spectral quality of the excitation beam at different wavelengths was also good and fairly consistent. However, the pulse duration of the OPA used in our measurements showed an increasing trend with pump wavelength, as can be observed in Figure S2 in the Supplemental Information. This is a technical limitation that is inherent to OPAs, which are also expected to have significantly diminished output power at longer pump wavelengths. The ZnTe detector crystal has high enough sensitivity but its 1-mm thickness limits the measured bandwidths to ∼2 THz. Both of these limitations do not change the conclusions drawn from our experiments and the overall work, as clarified with supporting details in the STAR Methods Section where the pump beam properties and the terahertz emission measurements are also described comprehensively.

## STAR★Methods

### Key resources table

**Table:** 

REAGENT or RESOURCE	SOURCE	IDENTIFIER
**Software and algorithms**		

OriginPro 9.0	OriginLab	http://www.originlab.com
Vienna *Ab-initio* Simulation Package (VASP)	VASP Software GmbH	http://www.vasp.at
Gnuplot	gnuplot	http://www.gnuplot.info

**Other**		

MgO (100) substrate	Crystal	http://www.crystal-gmbh.com/
Iron (Fe) target material	Praxair Electronics	MRCFFE0024268
Platinum (Pt) target material	Praxair Electronics	MRCFPT0025084

### Resource availability

#### Lead contact

Further information and requests for resources relevant to this work should be directed to and will be fulfilled by the lead contact, Valynn Katrine Mag-usara (valynn@ile.osaka-u.ac.jp).

#### Materials availability

This study did not generate new unique reagents.

### Experimental model and subject details

This study does not use experimental methods typical in the life sciences.

### Method details

#### Fe/Pt bilayer fabrication and characterization

The 2-nm Fe and 3-nm Pt thin films were epitaxial grown on a 500-μm-thick MgO (100) substrate by molecular beam epitaxy (MBE) technique inside an ultrahigh vacuum chamber with a base pressure of 3 × 10^−11^ mbar. First, the 1 × 1 cm^2^ substrate was prepared for the deposition of the Fe and Pt metallic layers by implementing a cleaning protocol, which involved heating at 600°C for 1 h and plasma-etching process using a 50–50% mix of Ar and O_2_ gas. Then, with the Fe beam oriented perpendicular to the substrate, the Fe thin film was deposited on the MgO at a rate of 0.05 Å/s, using a calibrated quartz crystal oscillator to *in-situ* monitor and control the process at 300°C growth temperature. The next deposition stage was the epitaxial growth of the Pt layer on top of the Fe layer at the same temperature. The thickness of each thin film on the MgO substrate was confirmed *ex-situ* by X-ray reflectivity (XRR) measurements and the structural quality of the Fe/Pt bilayer was investigated by high-resolution energy-filtered transmission electron microscopy (HR EF-TEM), a process which is described in detail in a previous work (Nenno et al., 2019) also on epitaxial grown Fe (2 nm)/Pt (3 nm) bilayers. The results of the HR EF-TEM revealed the interface quality of the fully epitaxial stress-free growth of the Fe/Pt bilayer when the deposition was done by MBE entirely at 300°C. Good epitaxial growth of Fe/Pt on MgO was also confirmed through X-ray diffraction measurements (XRD) (Torosyan et al., 2018). In another study, where the same deposition process and control parameters were also implemented in making spintronic bilayers with thicker Fe and Pt films, the XRD patterns revealed the epitaxy of Fe(100) on MgO(100) and Pt(100) on Fe(100) with the epitaxial relations described as Fe[001]||MgO[011] and Pt[011]||Fe[001], respectively (Torosyan et al., 2018; Papaioannou et al., 2013). The XRD results implied that the *fcc* lattice of the Pt grew on top of the *bcc* lattice of the Fe by rotating its *fcc* cell by 45° with respect to the Fe lattice, allowing for the overall high quality of epitaxial growth of Pt on Fe on MgO as clearly observed through HR EF-TEM.

#### Spintronic terahertz emission measurements

In this work, all THz emission measurements were carried out in ambient temperature (21 ± 0.1°C) and humidity (55 ± 0.5%) conditions using an in-house-built THz time-domain spectroscopy (THz-TDS) setup, as illustrated in Figure S1. The main laser source of the probe and pump beams was a Ti:sapphire regenerative amplifier system (Spitfire Ace, Spectra-Physics), which had output energy stability within 0.5% rms and, thus, was consistent in providing horizontal linearly polarized ∼70-fs pulses with 806-nm central wavelength at a repetition rate of 1 kHz. To generate the different pump wavelengths necessary for this study, an optical parametric amplifier (OPA) (TOPAS-Prime, Light Conversion) was used in tandem with the regenerative amplifier system. The low-noise performance reliability and excellent beam pointing stability of the regenerative amplifier were ideal in operating the OPA unit, which delivered a steady train of femtosecond pump pulses at every desired output wavelength to optically excite the Fe/Pt emitter. The spectral quality of the output beam of the OPA can be confirmed using Figure S3, which shows the spectra of the OPA at select wavelengths. The spectral shape looks overall quite good for each wavelength, and the full-width at half-maximum (FWHM) did not change significantly.

From the output window of the OPA laser system, the collimated pump beam passed through suitable optics, which directed it to a computer-controlled delay stage, an optical chopper, and, eventually, onto the emitter. The emitter was carefully mounted to ensure that the pump pulses would hit a fixed spot on the spintronic bilayer plane (Pt film side first) at normal incidence, with 5-mW average power and an effective spot diameter of 4 mm. Moreover, a constant ∼15-mT external magnetic field was applied vertically along the plane of the Fe film, which is also orthogonal to the direction of the pump beam, as shown in the schematic provided in Figure 1. This configuration for spintronic THz generation was integrated in the THz-TDS setup such that, regardless of the polarization and wavelength of the pump beam, the THz waves from the Fe/Pt bilayer would always radiate linearly p-polarized. While residual transmitted pump pulses were blocked, a set of parabolic mirrors collected the transmitted THz waves, for subsequent detection by electro-optic sampling (EOS) using a 1-mm-thick ZnTe (110) crystal, EOS optics, and balanced photodetector (PDB210A/M, ThorLabs) connected to a lock-in amplifier (SR830 DSP, Stanford Research Systems) and data acquisition system. So that only the THz signals from the emitter were picked up and recorded, the reference signal from the optical chopper controller, which was set to a frequency of 500 Hz (the frequency at which the optical pump beam was mechanically modulated by the optical chopper), was used as reference for the lock-in amplifier.

To detect the transmitted THz radiation by EOS, 806-nm optical pulses from the laser system were directed to the ZnTe, which allowed for THz detection upon the spatial overlapping and synchronous arrival of the THz radiation with the optical probe pulses in the electro-optic crystal. The time-domain waveforms of the THz signal were measured by monitoring the THz electric field-induced changes to the optical probe while varying the difference in arrival time between the THz radiation and the optical probe pulses. We note that the THz detection bandwidth of EO sampling with 1-mm thick ZnTe is around 2 THz. However, this limited frequency bandwidth of the measuring system has no influence on the conclusions drawn from the experiments.

To ensure that the THz-TDS setup was working well-optimized according to design, a 1-mm-thick ZnTe (110) was first used as the THz source. The ZnTe was optically excited at 806-nm wavelength by pump pulses hitting the crystal with 5-mW average power and 2.5-mm beam spot diameter. The THz emission of the ZnTe was measured and, right after, the same experiment was repeated using the Fe/Pt spintronic bilayer emitter for comparison, which is shown in Figure S4. The THz emission of the Fe/Pt on MgO, when irradiated with 806-nm pump beam with 2.5-mm-diameter spot size, is approximately 75% of that generated by the ZnTe crystal. After these preliminary measurements, the diameter of the pump beam spot was changed to 4 mm using a pre-installed iris diaphragm in front of the emitter mount. The properties of the collimated output beam of the OPA and the experimental configuration allowed for the beam spot size to be sufficiently controlled by a single aperture without risk of distortion. For all excitation wavelengths used in this work, the output beam diameter of the OPA (>1 cm) was large enough to keep the divergence insignificant over the propagation distance. The 4-mm pump beam spot diameter was found to be ideal because it was sufficiently smaller than the collimated output beam of the OPA. At the same time, it allowed for the average pump power to be at 5 mW for all measurements even when the output power of the OPA became most limited at 2700 nm. The pump beam spot size versus full beam size was additionally monitored by confirming that the average power through the 4-mm aperture stayed proportionally below the full beam power. For this purpose, a calibrated thermopile sensor that is suitable for the 200-nm to 20-μm wavelength range was used to measure the beam power. There would be no reason for any beam of smaller diameter to pass through the opening of the aperture because there was only one beam source for each target pump wavelength and the collimated output beam of the OPA was neither reshaped nor refocused prior to the aperture. Since neither the alignment nor the focusing of the collimated pump beam onto the emitter surface was changed, the spot size also cannot be smaller than the aperture of the iris diaphragm. To keep the pump power at 5 mW, a variable neutral density filter with 0.6-mm-thick glass substrate was used between the OPA output port and the iris diaphragm. This method of keeping the pump beam power constant with the 4-mm aperture mainly affected the optical path length between the pump and the gating beam for the EO sampling but not the pump pulse duration. By design, the variable neutral density filter attenuates the intensity of the laser beam without changing the other beam properties. With the overall configuration for THz generation and detection, it was convenient to change the THz source without changing the alignment of the THz optics and of the detector. In addition, the stability of the wavelength-tunable laser system guaranteed comparable experimental conditions even as the output wavelength was changed to investigate the pump wavelength dependence of the spintronic THz emission. As can be confirmed through the autocorrelation plots in Figure S2B and the spectra in Figure S3, the output beam of the OPA was consistently Gaussian, which also aptly describes the beam intensity profile.

The pump pulses with 800 nm–2000 nm wavelengths had pulse durations in the range of 50–70 fs and those with wavelengths above 2000 nm had >70-fs pulse duration, as shown in Figure S2. We note, however, that the different range of pulse durations between the sets of pump wavelengths below and above 2000 nm does not affect the conclusions drawn from our experiments and the over-all work.

Here, let us consider the influence of pump pulse duration to the THz emission efficiency. Since the THz emission is essentially proportional to the time derivative of the charge current, the pump pulse duration induces change in the charge current such that, with all other parameters kept constant, a slower change (due to longer pulse durations) of the charge current would mean lower amplitude of the THz emission due to lower time derivative values. With longer pulse durations, THz amplitudes and bandwidths are expected to decrease. However, there are important clues in perusing Figures 2, 3, 4, S2, and S5 that the observed decrease or increase in THz amplitude or in bandwidth in our experiments is not mainly due to the pulse duration. The influence of the pump pulse-width variation might be small since the observed THz pulse shape with different wavelengths, as shown in Figure 2A, are almost the same except in amplitude (see also Figure S5A). Moreover, both the time-domain THz waveforms and spectra in Figure 2 show that the THz emission of the Fe/Pt bilayer is better at 1550-nm optical excitation than at either 800 nm or 1000 nm but based on the measured pulse durations in Figure S2, the 1550-nm pump has 15-fs and 7-fs longer pulse durations than the 800-nm and 1000-nm pump beams, respectively. Similarly, it can be observed in Figures 3 and S5B that the THz generation at 2100 nm is better than at 2000 nm even though Figure S2 shows that the pulse duration of the 2100-nm pump is 16-fs longer than that of the 2000-nm pump. On the other hand, the THz generation by the 2000-nm pump, which has 5-fs longer pulse compared to the 1550-nm pump, exhibited a significant decrease in both time-domain amplitude and spectral content. In addition, the trend of the THz amplitudes remained consistent with that of the absorptance until the 2500-nm pump wavelength even though it can be deduced from the pulse duration vs. wavelength plot in Figure S2A that the pulse durations were already significantly increasing beyond 2000 nm. More importantly, in Figure 3 and even when the influence of the absorptance is already taken into account as shown in Figure 4, the THz amplitudes did not exhibit an increase then decrease in the 1500-nm to 2100-nm optical excitation region as they should have, considering the trend of the pulse widths in the range. The expected effects of longer pulse durations, such as wider THz pulse widths with decrease in THz amplitudes and spectral bandwidths were not consistently observed in this case. Clearly, the longer pulse widths of the low photon energy pump cannot be the primary reason for the observed decrease in THz amplitudes. Furthermore, there are both other experimental and theoretical methods to confirm the efficiency of the absorptance and the spin transport dynamics with respect to pump wavelengths and photon energies. Hence, we argue that the pulse durations, although should not be ignored, do not change the conclusions drawn from our experiments and overall work.

#### Absorptance calculations and measurements

To account for the influence of pump light absorption on the spintronic THz emission at different pump wavelengths, the absorptance of the epitaxial Fe/Pt bilayer was evaluated through calculations for a wide range of wavelengths and presented in Figure 3. The absorptance values were obtained by first determining the transmittance and reflectance values of the metallic heterostructure, then by computing for the absorptance values using α = 1 – τ – ρ, where α is the absorptance, τ is the transmittance (the ratio of transmitted optical power to the incident optical power), and ρ is the reflectance (the ratio of the reflected optical power to the incident optical power). The formulae for calculating the pump wavelength-dependent transmittance and reflectance are found in literature (Tomlin, 1972; Barybin and Shapovalov, 2010), and allow for proper consideration of parameters and conditions that are consistent with the experimental configuration for the optical excitation of the spintronic THz emitter. The optical constants, which are necessary inputs for the computation, are based on published information on the wavelength-dependent refractive indices and absorption coefficients of Pt (Rakic et al., 1998) and Fe (Ordal et al., 1988; Johnson and Christy, 1974; Werner et al., 2009) as well as on the refractive indices of air (Ciddor, 1996; Mathar, 2007) and MgO (Stephens and Malitson, 1952). The calculations take into account the interference of transmitted and reflected waves by the interfaces in a multilayer system, where both the Pt and Fe thin films are the main parts of a 4-layer stack consisting of the input medium (ambient air), the double film stack with 3-nm Pt and 2-nm Fe, and the MgO substrate which is the output medium.

The absorptance curve shown in Figure 4 inset was derived from the Fe/Pt transmittance and reflectance, as measured by Fourier-transform infrared (FTIR) spectroscopy using a deuterated L-alanine-doped triglycine sulphate (DLaTGS) detector. The plots of the measured transmittance and reflectance are provided in the Supplemental Information as Figure S6.

#### Spin-polarized density functional theory (SDFT) calculations

The Fe/Pt bilayer is modeled by a 6-layer slab of Fe(001) and a 6-layer slab of pseudomorphic Pt(001). Since the Fe/Pt bilayer in this work is epitaxial grown, then we also laid the Pt(001) pseudomorphically onto the Fe(001) slab. No intercalation of atoms is conducted at the interface because the spin-up reflectivity for both clean and disordered interfaces are the same (Lu et al., 2020). Thus, in the atomic modelling, the use of the former would suffice. The atomic structure and the electronic structure (LDOS) are obtained using SDFT, which was implemented in Vienna *Ab-initio* Simulation Package (VASP) (Kresse and Hafner, 1993; Kresse and Furthmuller, 1996). The ion-electron interaction was treated using projector augmented wave (PAW) method (Blochl, 1994), while the exchange and correlation was described by the generalized gradient approximation (GGA) based on the Perdew, Burke and Ernzerhof (PBE) functional (Perdew et al., 1996). Plane-wave basis set with a cut-off energy of 300 eV was used. Brillouin zone integrations were performed on a grid of 4 × 4 × 1 Monkhorst-Pack k-points with a smearing of Methfessel-Paxton method (Methfessel and Paxton, 1989) for the optimization calculations while the tetrahedron method with a larger k-point grid of 11 × 11 × 1 was used for more accurate calculation of LDOS. In this work, we calculated the LDOS for the Fe side, that is the DOS for Fe atoms at the interface and at the bulk region. All the 12 layers of the Fe/Pt were optimized by full ionic and electronic relaxations using conjugate gradient algorithm and block-Davidson algorithm, respectively. Structure optimization calculations were stopped when the Hellmann-Feynman forces on the atoms are less than 0.003 eV/Å. The above calculation methods yielded lattice parameter values for Fe and Pt bulk systems in agreement with experiments (Kittel, 2005), as shown in Table S1. The spin magnetic moment of Fe bulk was found to be 2.224 μ_B_, also in excellent agreement with the experimental value of 2.2 μ_B_ (Danan et al., 1968). Table S2 shows the Fe-Pt interlayer distance along z, *d* and Fe-Pt bond length, *L* in comparison with the bulk counterparts (Janthon et al., 2014). In general, we can see that the Fe-Pt interface distances are intermediate of Fe and Pt bulk distances, as expected.

### Quantification and statistical analysis

The raw simulation data were generated by VASP software and graphed using gnuplot. All other data plots for the figures in the main text and Supplemental Information were produced by OriginPro using the raw experimental data and, for theoretical absorptance of Fe/Pt, using published data. Information about the error bars, when they are included in the figures, are provided in the legends.

## Data Availability

The data reported in this paper are available from the lead contact upon reasonable request. This paper does not report original code. Any additional information required to reanalyze the data reported in this paper is available from the lead contact upon request.
