# Supplementary material for: Optimum excitation wavelength and photon energy threshold for spintronic terahertz emission from Fe/Pt bilayer
Source: iScience. 2022 Jun 16;25(7):104615. doi: 10.1016/j.isci.2022.104615 (PMC9253697; doi:10.1016/j.isci.2022.104615)
Supplement: Document S1. Figures S1–S6 and Tables S1 and S2 [file mmc1.pdf]

iScience, Volume *25*

Supplemental information

Optimum excitation wavelength and photon energy threshold for spintronic terahertz emission from Fe/Pt bilayer

Valynn Katrine Mag-usara, Mary Clare Escaño, Christopher E. Petoukhoff, Garik Torosyan, Laura Scheuer, Julien Madéo, Jessica Afalla, Miezel L. Talara, Joselito E. Muldera, Hideaki Kitahara, David R. Bacon, Makoto Nakajima, Keshav Dani, Evangelos Th. Papaioannou, René Beigang, and Masahiko Tani

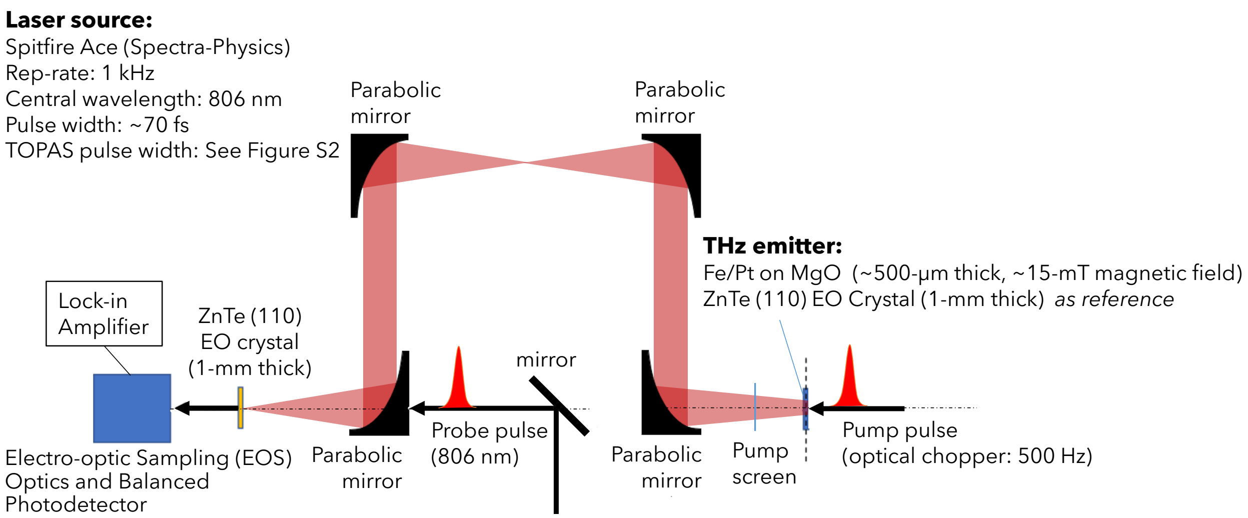

Figure S1. Schematic of the THz time-domain spectroscopy setup with wavelength tunable laser source used to evaluate the wavelength dependence of the spintronic THz emission of the Fe/Pt bilayer. Related to STAR Methods.

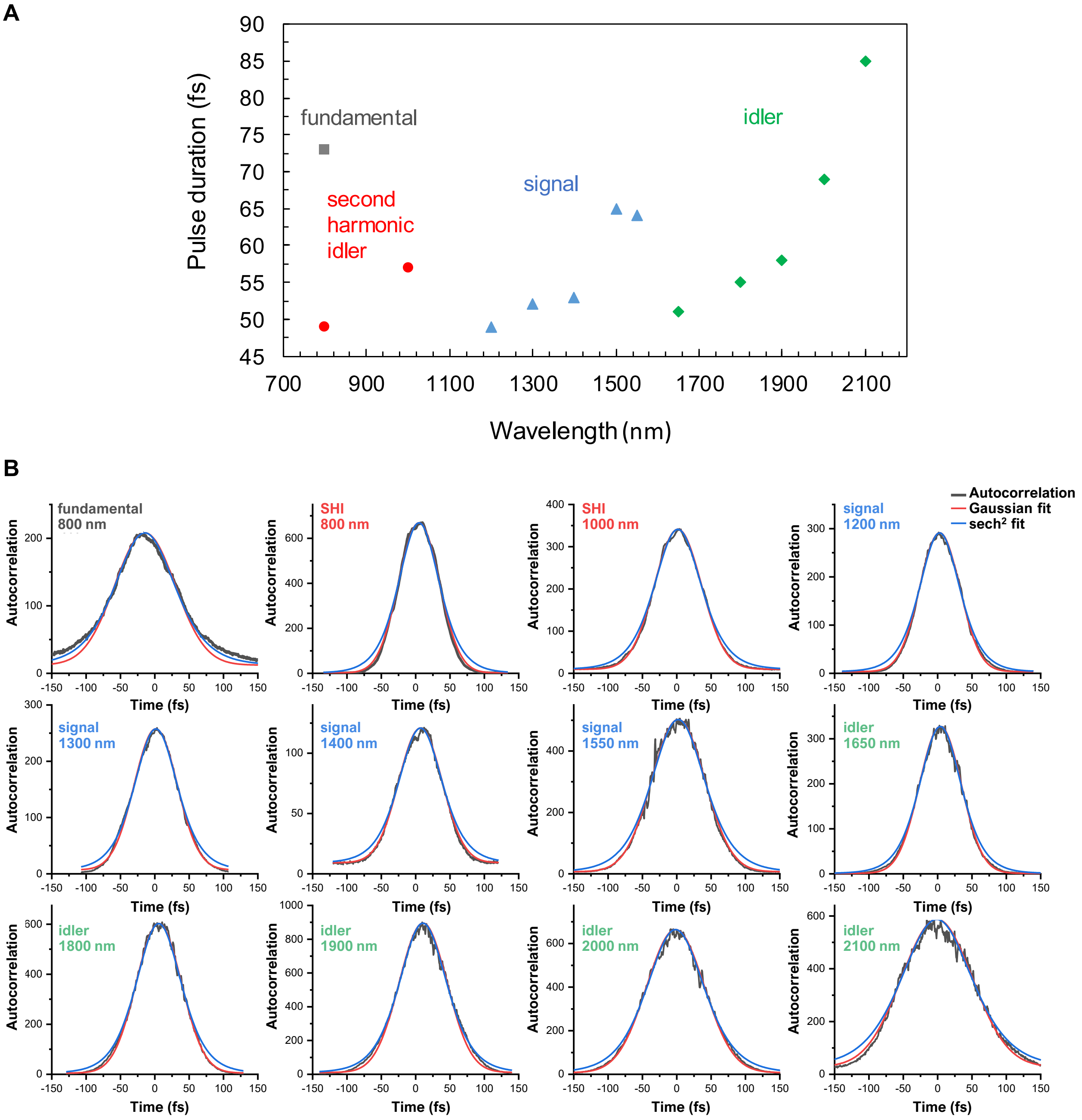

Figure S2. (A) Gaussian pulse duration vs. output wavelength of the optical parametric amplifier (TOPAS-Prime, Light Conversion) and (B) autocorrelation measurements with Gaussian fit at each wavelength. Related to Limitations of the study and STAR Methods.

The interactions (SHI: second harmonic of idler, signal, idler) are also indicated. Femto Easy Fast Frequency-resolved Optical Grating (FROG) was used to measure the autocorrelation of the TOPAS output at the wavelengths used in the paper, from 800 to 2100 nm. The maximum wavelength of the Femto Easy Fast FROG is 2150 nm, which limits the range of pulse duration measurements from 800 nm to 2100 nm only. The autocorrelation curves shown in (B) were measured just before the terahertz emitter using the same beam spot size (4-mm diameter) and average power (5 mW) used for the terahertz emission measurements. The autocorrelation of the fundamental output (from the main laser source: Spitfire Ace, Spectra-Physics) is included for reference.

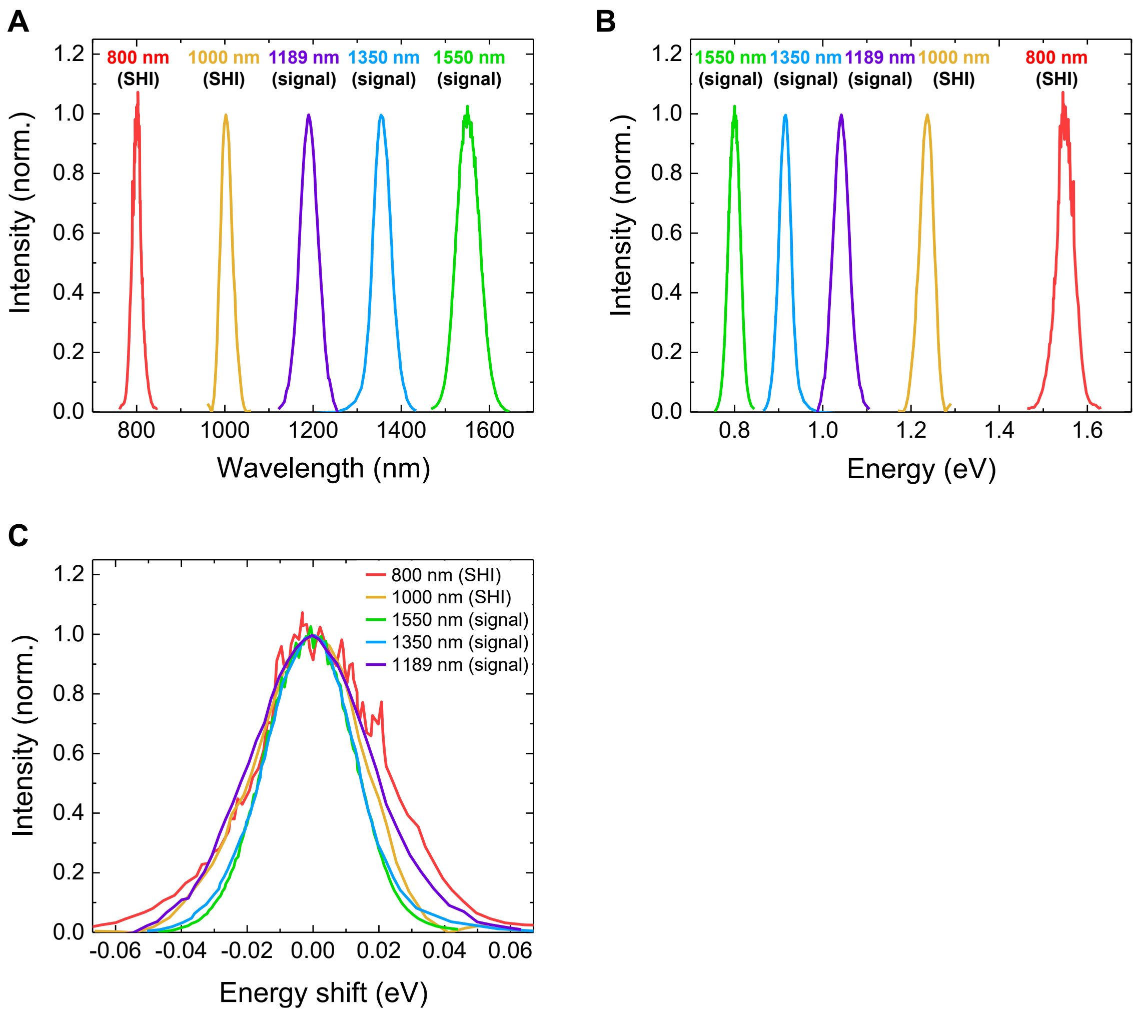

Figure S3. The spectra of the output beam of the optical parametric amplifier (TOPAS-Prime, Light Conversion) at select wavelengths. Related to STAR Methods.

The spectra are shown together on the same (A) wavelength and (B) energy scales. The representative spectra have central wavelengths at 800 nm (SHI: second harmonic of idler), 1000 nm (SHI), 1189 nm (signal), 1350 nm (signal), and 1550 nm (signal). Only the 800-nm spectrum was obtained with a different spectrometer (Ocean Optics) due to wavelength range limitations (900 to 1700 nm) of the Hamamatsu Photonics spectrometer. The idler that was generated with the 1350-nm signal beam was used as the 2000-nm excitation beam for terahertz measurements, and the second harmonic of the idler was used as the 1000-nm pump beam for terahertz generation. In the same manner, the idler generated with the 1189-nm signal beam was used as the excitation beam for terahertz measurements with 2500-nm pump. The comparison in (C) shows that the spectral quality of the excitation beam was generally good and consistent.

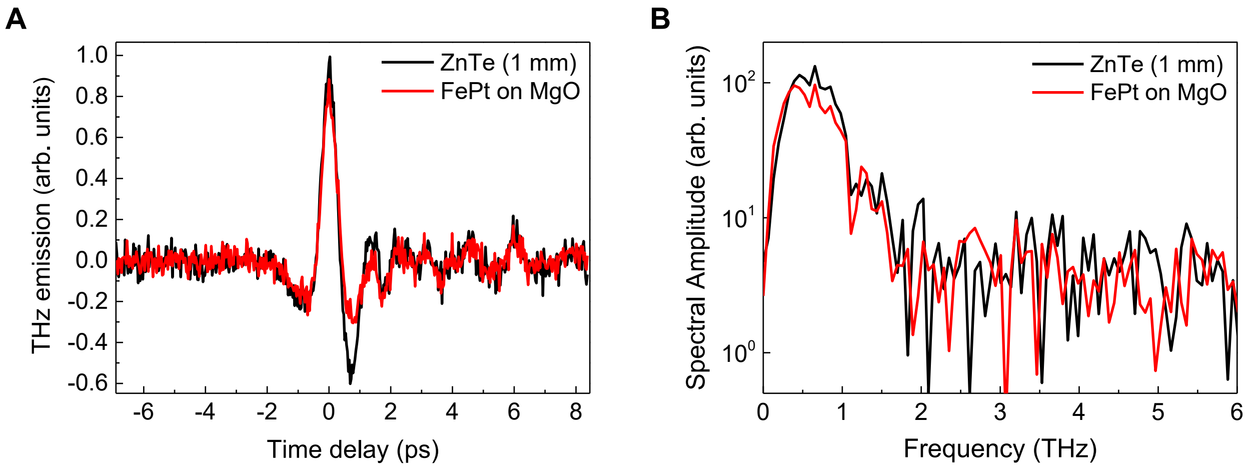

Figure S4. Comparison of THz emission from 1-mm thick ZnTe (110) electro-optic emitter and the Fe/Pt spintronic emitter at 806-nm excitation using the same pump pulse parameters and THz detection conditions. Related to STAR Methods.

Shown are the (A) THz time-domain waveforms and (B) corresponding spectra. At the same pump fluence, the THz emission of the Fe/Pt on MgO is approximately 75% of that generated by the ZnTe crystal. The frequency bandwidths are consistent with the THz detection bandwidth of EO sampling using 1-mm thick ZnTe crystal, which is limited to around 2 THz. Since the measurements were done under ambient room conditions with the relative humidity at 55 +/- 0.5%, the influence of water vapor absorption can be observed as oscillations after each main pulse in (A) and as characteristic water absorption bands in (B).

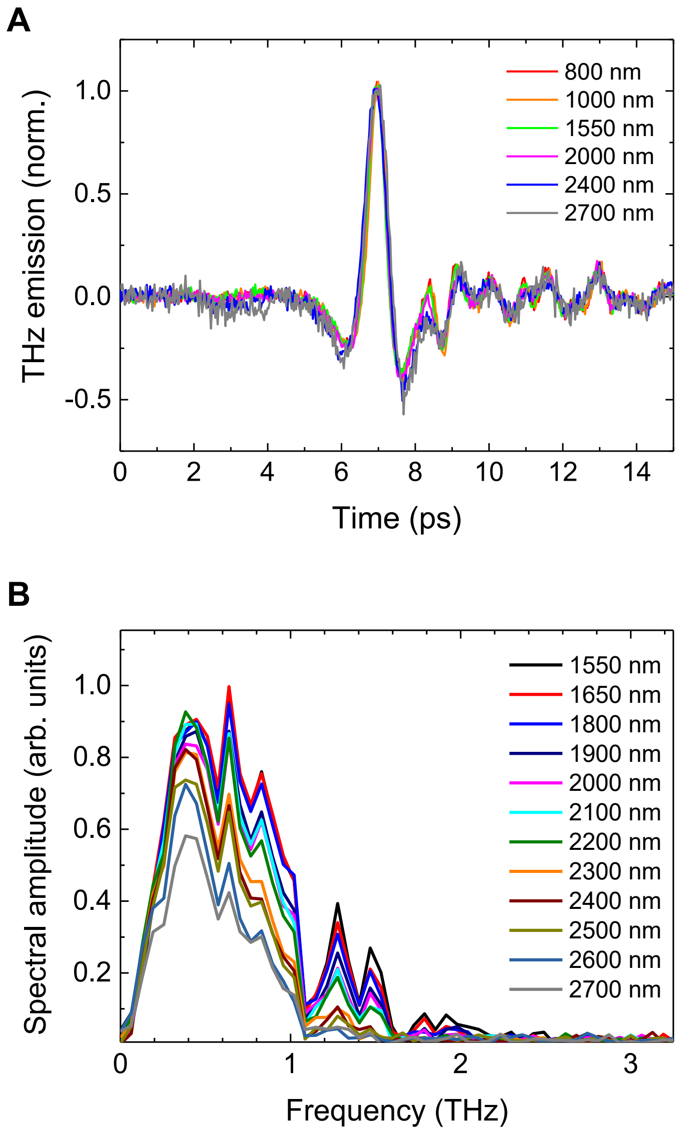

Figure S5. (A) Normalized representative waveforms of the terahertz emission from the Fe/Pt spintronic bilayer; and (B) terahertz emission spectra of the Fe/Pt bilayer at higher pump wavelengths. Related to Figure 2 and STAR Methods.

The beam pointing, average pump power (5 mW), and pump beam spot diameter (4-mm) on the Fe/Pt spintronic terahertz emitter were kept constant. Based on the normalized waveforms in (A), there is no strong indication that the different pulse durations of the excitation beams significantly influence the terahertz emission.

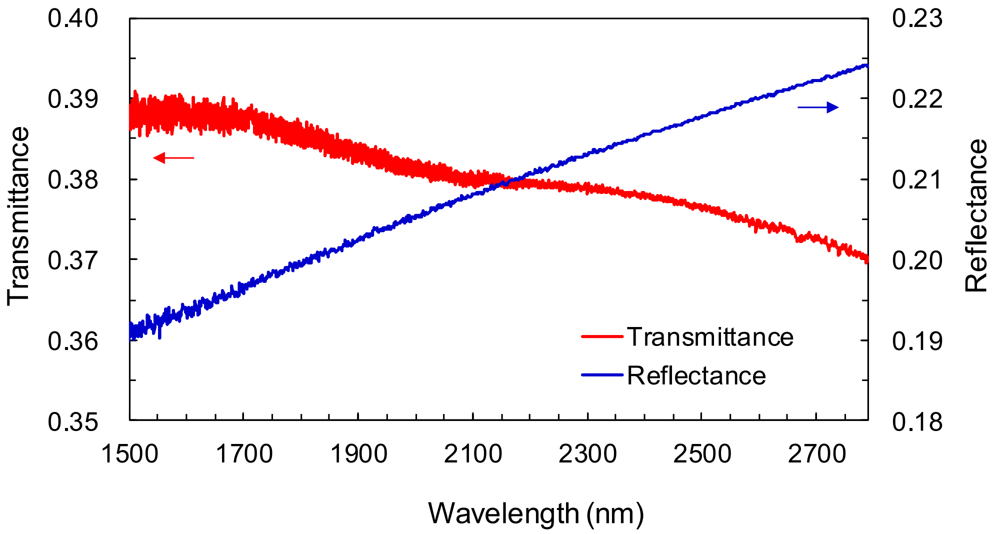

Figure S6. Transmittance (red) and reflectance (blue) of the Fe/Pt bilayer, as measured by Fourier-transform infrared (FTIR) spectroscopy using a deuterated L-alanine-doped triglycine sulphate (DLaTGS) detector. Related to STAR Methods.

Table S1. Lattice parameter, *a* of bcc Fe bulk and fcc Pt bulk given in angstrom, magnetic moment, *S* of Fe bulk (per atom) given in bohr magneton, μ_B in comparison with experimental values. Related to STAR Methods.

		This calc.	Exp.
bcc Fe (bulk)	*a* (Å)	2.830	2.87 (Kittel, 2005)
	S (μ_B)	2.224	2.2 (Danan, Herr, and Meyer, 1968)
fcc Pt (bulk)	*a* (Å)	3.970	3.920 (Kittel, 2005)

Table S2. Bond length, *L* and interlayer distance along z, *d* at the Fe/Pt interface and the corresponding calculated and experimental bulk values are also given. Related to Figure 5 and STAR Methods.

	Distances (Å)	This calc.	Exp. (Janthon et al., 2014)
Interface	Fe-Pt *d*	1.627	-
	Fe-Pt *L*	2.579	-
bcc Fe (bulk)	Fe-Fe *d*	1.415	1.435
	Fe-Fe *L* (shortest)	2.451	2.460
fcc Pt (bulk)	Pt-Pt *d*	1.985	1.960
	Pt-Pt *L* (shortest)	2.807	2.811
